# MoS_2_-Decorated/Integrated Carbon Fiber: Phase Engineering Well-Regulated Microwave Absorber

**DOI:** 10.1007/s40820-021-00646-y

**Published:** 2021-04-27

**Authors:** Jing Yan, Ying Huang, Xiangyong Zhang, Xin Gong, Chen Chen, Guangdi Nie, Xudong Liu, Panbo Liu

**Affiliations:** 1 MOE Key Laboratory of Material Physics and Chemistry Under Extraodinary Conditions School of Chemistry and Chemical Engineering, Ministry of Education, Northwestern Polytechnical University, Xi’an, 710072 People’s Republic of China; 2 School of Materials Science and Engineering, Central South University, Changsha, 410083 People’s Republic of China; 3 Institute of Flexible Electronics, Northwestern Polytechnical University, Xi’an, 710072 People’s Republic of China; 4 Industrial Research Institute of Nonwovens and Technical Textiles, College of Textiles and Clothing, Qingdao University, Qingdao, 266071 People’s Republic of China

**Keywords:** Phase engineering, Electromagnetic wave absorber, 1T/2H MoS_2_, 2H MoS_2_, Flexible film

## Abstract

Phase engineering is an important strategy to modulate the electronic structure of molybdenum disulfide (MoS_2_). MoS_2_-based composites are usually used for the electromagnetic wave (EMW) absorber, but the effect of different phases on the EMW absorbing performance, such as 1T and 2H phase, is still not studied. In this work, micro-1T/2H MoS_2_ is achieved via a facile one-step hydrothermal route, in which the 1T phase is induced by the intercalation of guest molecules and ions. The EMW absorption mechanism of single MoS_2_ is revealed by presenting a comparative study between 1T/2H MoS_2_ and 2H MoS_2_. As a result, 1T/2H MoS_2_ with the matrix loading of 15% exhibits excellent microwave absorption property than 2H MoS_2_. Furthermore, taking the advantage of 1T/2H MoS_2_, a flexible EMW absorbers that ultrathin 1T/2H MoS_2_ grown on the carbon fiber also performs outstanding performance only with the matrix loading of 5%. This work offers necessary reference to improve microwave absorption performance by phase engineering and design a new type of flexible electromagnetic wave absorption material to apply for the portable microwave absorption electronic devices.

**Supplementary Information:**

The online version contains supplementary material available at 10.1007/s40820-021-00646-y.

## Highlights

- A facile one‐step hydrothermal method for producing gram‐scale 1T@2H-MoS_2_ by imbedding the guest molecules and ions was developed.
- The influence of different MoS_2_ phase for electromagnetic absorbing properties was explored by analyzing electromagnetic parameters of 1T/2H MoS_2_ and 2H MoS_2_ with 50%, 40%, 30%, 20%, 15%, and 10% filler loading.
- Taking the advantage of 1T/2H MoS_2_, the flexible CF@1T/2H MoS_2_ was also synthesized to mind the request of flexible portable microwave absorption electronic devices.

**Supplementary Information:**

The online version contains supplementary material available at 10.1007/s40820-021-00646-y.

## Graphical abstract

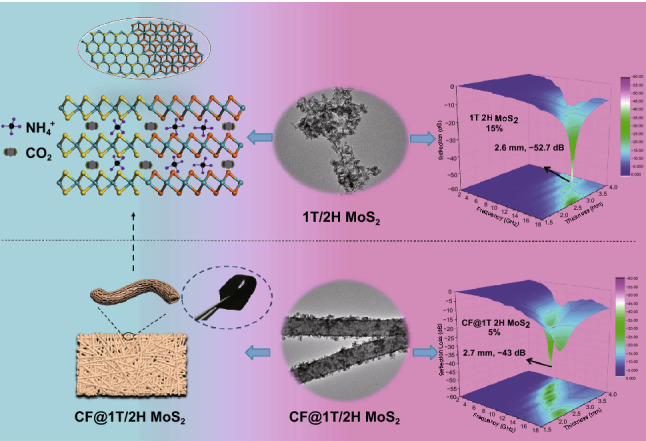

## Introduction

With the rapid development of electronic information technology, electromagnetic wave exists everywhere in our environment that not only interferes with the electromagnetic control system and negates the effects of equipment, but also harms the physical and mental health of human beings. Therefore, it is of great significance to develop absorbing materials with electromagnetic wave absorbing ability that can both mind the requirements of “wide, thin, light and strong” [1–4]. Moreover, the miniaturization and integration of electronic circuits and components put forward the request of flexible portable microwave absorption electronic devices [5, 6].

Two-dimension (2D) materials, such as reduced graphene oxide (RGO) [7–10], MoS_2_ [11–15], and new member Mxene [16–19], are usually applied as microwave absorber owing to their high specific surface area and abundant functional groups as well as defects, which will enhance the propagation paths of incident electromagnetic wave (EMW) inside absorbers by scattering effect and increase the loss through polarization relaxation loss. As one of the 2D material, MoS_2_ has adjustable electrical property and can change between insulators and semiconductor metals. It has important applications in many fields such as optoelectronics [20, 21], secondary batteries [22, 23], and catalysis [24, 25]. In addition, MoS_2_ also has been proved to be an effective dielectric-type EMW absorbing material. As we know, MoS_2_ exists in various phase forms, such as 2H, 1T, and 3R. The natural MoS_2_ usually exists in the form of 2H (hexagonal) phase that the stacking sequence is AbA, BaB (the capital and lower case letters denote chalcogen and metal atoms, respectively), showing an adjustable band gap of 1.3–1.9 eV and presenting semiconductor properties. However, the inherent low conductivity loss would limit its further practical application in microwave absorption to some extent. The octahedral coordination of 1T (triangle)-MoS_2_ structure has metallic property that the stacking sequence is AbC, AbC, and belongs to metastable structure, but it shows a high electrical conductivity [26]. Therefore, combined with above advantages, the mixed phase MoS_2_ (1T@2H-MoS_2_) would have the great application potential in the field of microwave absorption [27].

Up to now, many researches about 2H-MoS_2_ applied for EMW absorbing field have been reported [28]. Cao et al. [29] investigated the EMW absorption properties of few-layered pure MoS_2_ nanosheets that prepared by a top-down exfoliation method. The optimum electromagnetic absorbing performance parameters of MoS_2_-NS/wax with 60% loading are −38.42 dB, 2.4 mm. Though the single 2H MoS_2_ exhibited good EMW absorption performance, the sample in sample-paraffin needs to fill in a high proportion, this will undoubtedly limit their practical application. There already have a few advances about 1T@2H-MoS_2_ as one of the components to EMW absorbing. Liu et al. [30] designed a 3D carbon foam/1T@2H-MoS_2_ composites, which had the maximum reflection loss of −45.88 dB. Moreover, Che et al. [31] planted the 1T@2H-MoS_2_ into RGO via ammonia insert and high-temperature annealing of 2H-MoS_2_/RGO, and this composite exhibited the excellent EMW absorption ability with the sample mass ratio of 30% in sample-paraffin. These researches mainly focus on the 1T@2H-MoS_2_-based composites, not involve pure 1T@2H-MoS_2_, let alone profound explore the influence of different MoS_2_ phase for electromagnetic absorbing properties.

In this work, we try to prepare two type phases of MoS_2_ via a simple synthesis method and compare the electromagnetic parameters corresponding to different phases of MoS_2_, thus obtain the excellent EMW absorber by analyzing the EMW absorbing mechanism. Normally, the transformation of MoS_2_ from 2H to 1T can be achieved by chemical exfoliation or substitutional doping [32, 33], which are complex and low yield. Herein, we developed a facile one-step hydrothermal method for producing gram-scale 1T@2H-MoS_2_ by imbedding the guest molecules and ions. The 2H-MoS_2_ is obtained by annealing treatment of 1T@2H-MoS_2_. The results show that 2H MoS_2_ and 1T@2H-MoS_2_ both can effectively EMW absorbing. The synergistic effect between 1T phase and 2H phase of 1T@2H-MoS_2_ in EMW absorbing can further improve the dielectric loss, which makes single 1T@2H-MoS_2_ has a great application prospect in the field of EMW absorbing. In addition, a flexible EMW absorbers that ultrathin 1T/2H MoS_2_ grown on the carbon fiber (CF) by using the same method except the adding of CF, which performs outstanding performance only with the matrix loading of 5%, again prove the significance of this work.

## Experimental Section

### Synthesis of 1T/2H MoS_2_

The (NH_4_)_6_Mo_7_O_24_·H_2_O (0.88 g) and CH_3_CSNH_2_ (0.9 g) were ultrasonic dissolved in 50 mL water, and then 1.98 g NH_4_HCO_3_ was added and stirred for 30 min to form a uniform liquid. The above solution was transferred into the 100-mL hydrothermal synthesis reactor and heated to 200 °C for 13 h. After cooling to room temperature, the black sediments were collected and washed with deionized water and ethanol for further use. The final product was obtained by drying in the vacuum oven for 12 h.

### Synthesis of 2H MoS_2_

The above 1T/2H MoS_2_ was placed in an argon tube furnace and heated to 400 °C with a heating rate of 10 °C min^−1^ for 2 h to obtain 2H MoS_2_.

### Synthesis of CF@1T/2H MoS_2_ and CF@2H MoS_2_

The CF is obtained via a simple electrospinning method, and the detailed process is in the supporting information [34]. The synthesis process of CF@1T/2H MoS_2_ and CF@2H MoS_2_ is the same as 1T/2H MoS_2_ and 2H MoS_2_ except adding the 0.5 g CF after the solution is transferred to hydrothermal synthesis reactor.

## Results and Discussion

### Composition and Structure

**Scheme 1:**
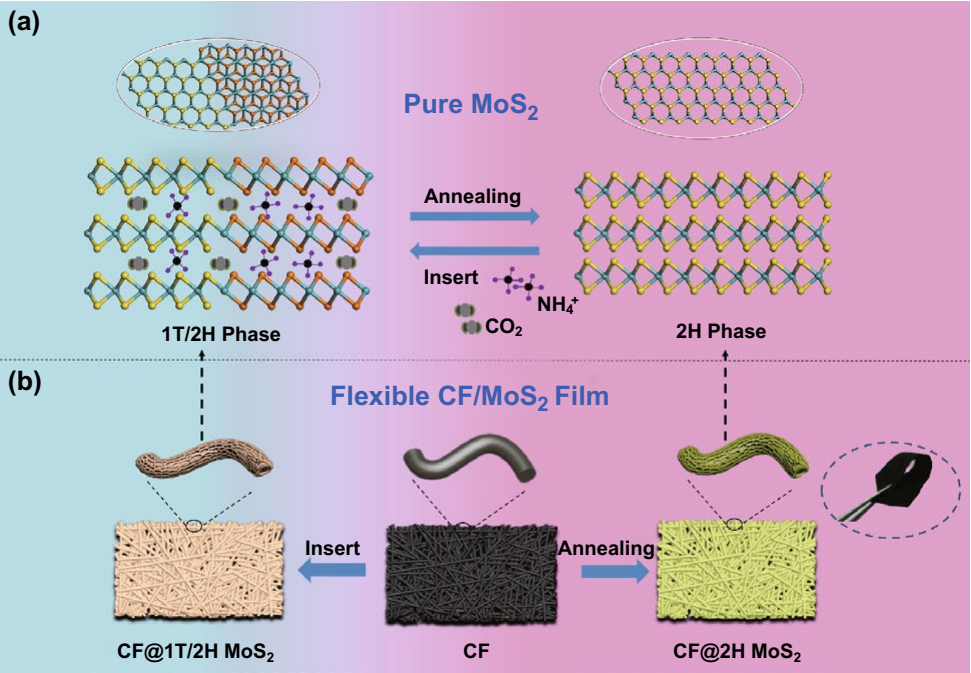
Schematic drawings illustrating the fabrication process of single MoS_2_ (1T/2H MoS_2_ and 2H MoS_2_) and flexible CF/MoS_2_ film (CF@1T/2H MoS_2_ and CF@2H MoS_2_)

The schematic diagram of single MoS_2_ is shown in Scheme 1a. The 1T/2H MoS_2_ is successfully fabricated by hydrothermal method. As a guest, the ammonium bicarbonate decomposes into small molecules and ions such as NH^4+^, H_2_O, and CO_2_, which are inserted into the layered structure of MoS_2_ to form 1T/2H phase polyphase MoS_2_, consisting of 1T phase and 2H phase. The electrical conductivity of 1T/2H phase is greatly improved owning to the existence of the 1T phase. Furthermore, the combination with 2H phase helps to stabilize the metastable 1T phase and avoids the re-accumulation and transition to 2H phase. The transition from 1T/2H MoS_2_ to 2H MoS_2_ is by annealing treatment. The synthesis principle of CF@1T/2H MoS_2_ is similar to that of 1T/2H MoS_2_, as shown in Scheme 1b. Considering the advantages of 1T/2H MoS_2_, the carbon fiber is chosen as a flexible substrate to obtain high-performance flexible EMW absorbing film.

**Fig. 1:**
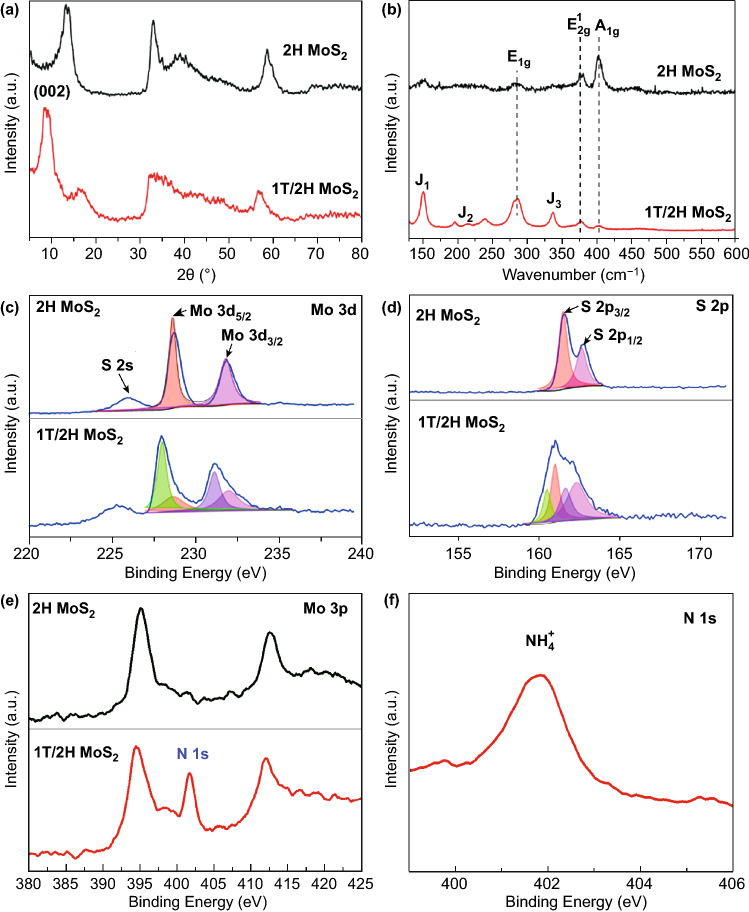
**a** XRD patterns, **b** XPS spectra, **c** Mo 3d spectra, **d** S 2p spectra, **e** Mo 3p spectra of 1T/2H MoS_2_ and 2H MoS_2_. **f** N 1s spectra of 1T/2H MoS_2_

The X-ray diffraction (XRD) patterns of MoS_2_ samples with different phases are shown in Fig. 1a. Obviously, the 1T/2H MoS_2_ and 2H MoS_2_ have different XRD patterns. The (002) crystal-peaks value of 2H MoS_2_ located at 14.4°, corresponds to the standard 2H phase bulk MoS_2_ (PDF card #75-1539) [35, 36]. As for 1T/2H MoS_2_, the corresponding (002) crystal peaks are situated at 2theta = 9.5° and 15.9°, and the *d* spacing difference between two (002) peaks proves the interlayer expansion. To further analyze the sample structure, Raman spectroscopy is introduced between 100 and 600 cm^−1^, as shown in Fig. 1b. The intensity ratio of peak *E*_1g_, *E*_2g_^1^, and *A*_1g_ is situated at 284.7, 381.7, and 403.3, respectively. However, the intensity of *E*_2g_^1^ and *A*_1g_ peaks over 1T/2H MoS_2_ is greatly decreased because of less 2H phase and worse crystallinity. As for 1T/2H MoS_2_, the additional strong peaks at 150.3 (J1), 215.6 (J2), and 336.8 (J3) cm^−1^ are observed, suggesting the formation of 1T phase MoS_2_. After annealing, the J1, J2, and J3 peaks of the 1T phase become very weak, and *E*_1g_, *E*_2g_^1^, and *A*_1g_ peaks of the 2H phase become more significant, indicating that the 1T phase is successfully converted to the stable 2H phase. X-ray photoelectron spectrometer (XPS) displays the element content on the material surface. In Fig. 1c, the two peaks located at around 229 and 232 eV correspond to the spectra of Mo 3d_5/2_ and Mo 3d_3/2_, clarifying the existence of 2H phase [37]. The peak at 226 eV corresponds to the spectra of S 2 s. Moreover, as for 1T/2H MoS_2_, two other peaks around 228 and 231 eV are observed, which have the 1 eV shift compared to that of 2H MoS_2_, proving the presence of the metallic 1T phase [38, 39]. Similarly, the two peaks can be observed at around 162.7 and 161.6 eV corresponding to the spectra of S 2p_1/2_ and S 2p_3/2_ (Fig. 1d). However, two additional peaks are found to shift to lower binding energies at around 161 and 160.5 eV, which once again suggests the presence of metallic 1T phase [39]. The content of 1T phase is estimated to be 61% by calculating the peak area. In Fig. 1e, the N 1 s in the Mo 3p spectra of 1T/2H MoS_2_ demonstrates the presence of the N element in 1T/2H MoS_2_. But no N element can be detected after annealing in 2H MoS_2_. The peak at around 402 eV corresponding to the spectra of the N 1 s in Fig. 1f should be attributed to the intercalation of NH^4+^.

**Fig. 2:**
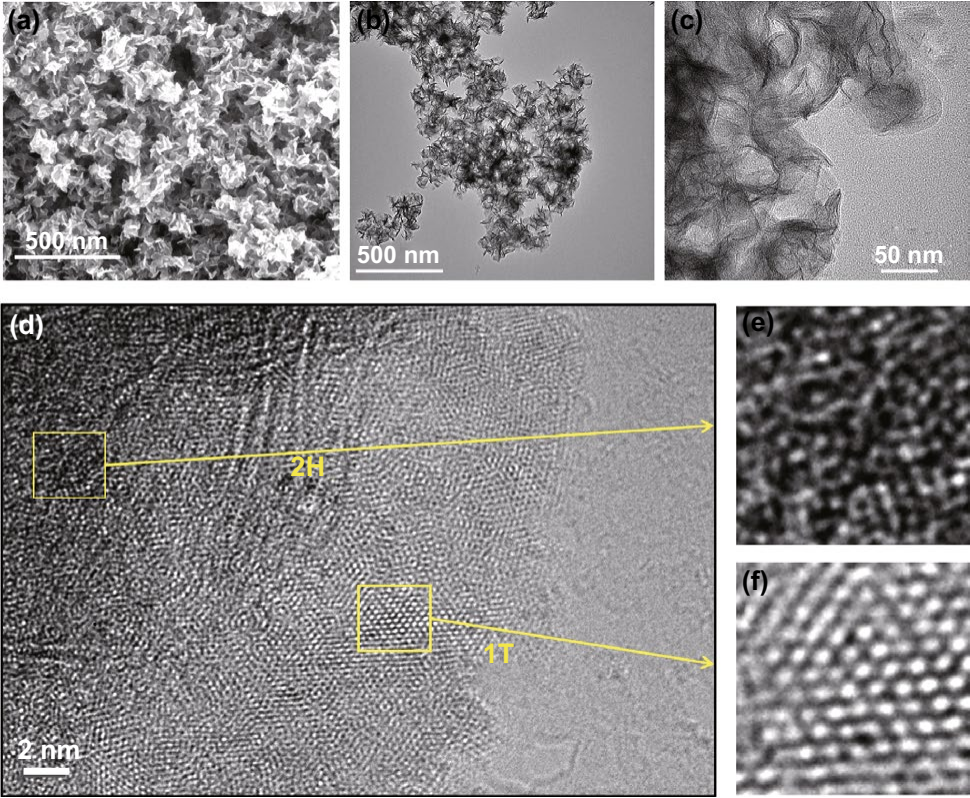
**a** SEM image, **b**, **c** TEM image, **d** HRTEM images of 1T/2H MoS_2_. **e**, **f** Enlarged images of the selected yellow area

Figure 2 shows the SEM and TEM images of synthesized 1T/2H MoS_2_. The SEM image (Fig. 2a) demonstrates that the 1T/2H MoS_2_ is made up of an infinite number of tiny nanosheets, which is more clearly revealed by the TEM image in Fig. 2b, c. The crosswise dimension of each lamellar is approximately 80 nm. The high-resolution TEM image (Fig. 2d) confirms the co-existence of the trigonal prismatic 2H phase and the octahedral 1T phase in 1T/2H MoS_2_. Furthermore, the lateral heterostructures of 1T (Fig. 2e) and 2H (Fig. 2f) phases could also be clearly visualized by zooming in the selected area of Fig. 2d. The element mapping images and EDX (Fig. S1) of 1T/2H MoS_2_ demonstrate the uniform distribution of Mo and S elements. The N in the mapping of 1T/2H MoS_2_ also demonstrates the presence of the N element in 1T/2H MoS_2_, which is consistent with the XPS results. After annealing, the absence of N of the annealed 2H MoS_2_ as revealed by Fig. S2, the morphology and microstructure of 2H MoS_2_ are observed as shown in Fig. S3. On macroscopic view, the 2H MoS_2_ remains the same morphology of 1T/2H MoS_2_, but only the 2H phase can be find in the zoom HRTEM Fig. S3d, which demonstrates 1T phase is transformed into 2H phase under annealing condition completely.

**Fig. 3:**
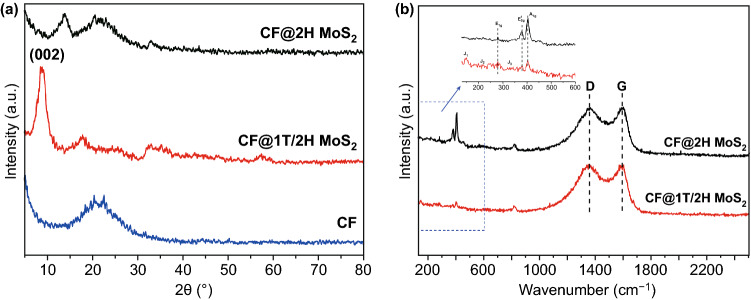
**a** XRD patterns, **b** Raman spectroscopy of CF@1T/2H MoS_2_ and CF@2H MoS_2_

To meet the demand of portable microwave absorption electronic devices, high-performance flexible film absorbers are urgently needed to be developed. Inspired by the synthetic methods of single 1T/2H MoS_2_ and 2H MoS_2_, the CF is added to the experiment as a substrate to obtain the flexible CF@1T/2H MoS_2_ and CF@2H MoS_2_ films. The XRD pattern and Raman spectrum of sample are shown in Fig. 3. In Fig. 3a, the CF@1T/2H MoS_2_ and CF@2H MoS_2_ have the same peaks as previous 1T/2H MoS_2_ and CF@2H MoS_2_ except the peak of carbon. As for Fig. 3b, the intensity ratio of peak D and G, situated at 1350 and 1580 cm^−1^, respectively, the characteristic peak of carbon material, can reflect the presence of carbon fiber. In this figure, the peaks of MoS_2_ are not obvious because the intensity of D peak and G peak is too high. After zooming the area of 150–600 cm^−1^, the *E*_1g_, *E*_2g_^1^, *A*_1g_ in both CF@1T/2H MoS_2_ and CF@2H MoS_2_, the J1, J2, J3 peaks in CF@1T/2H MoS_2_ are the same as those in Fig. 1b.

**Fig. 4:**
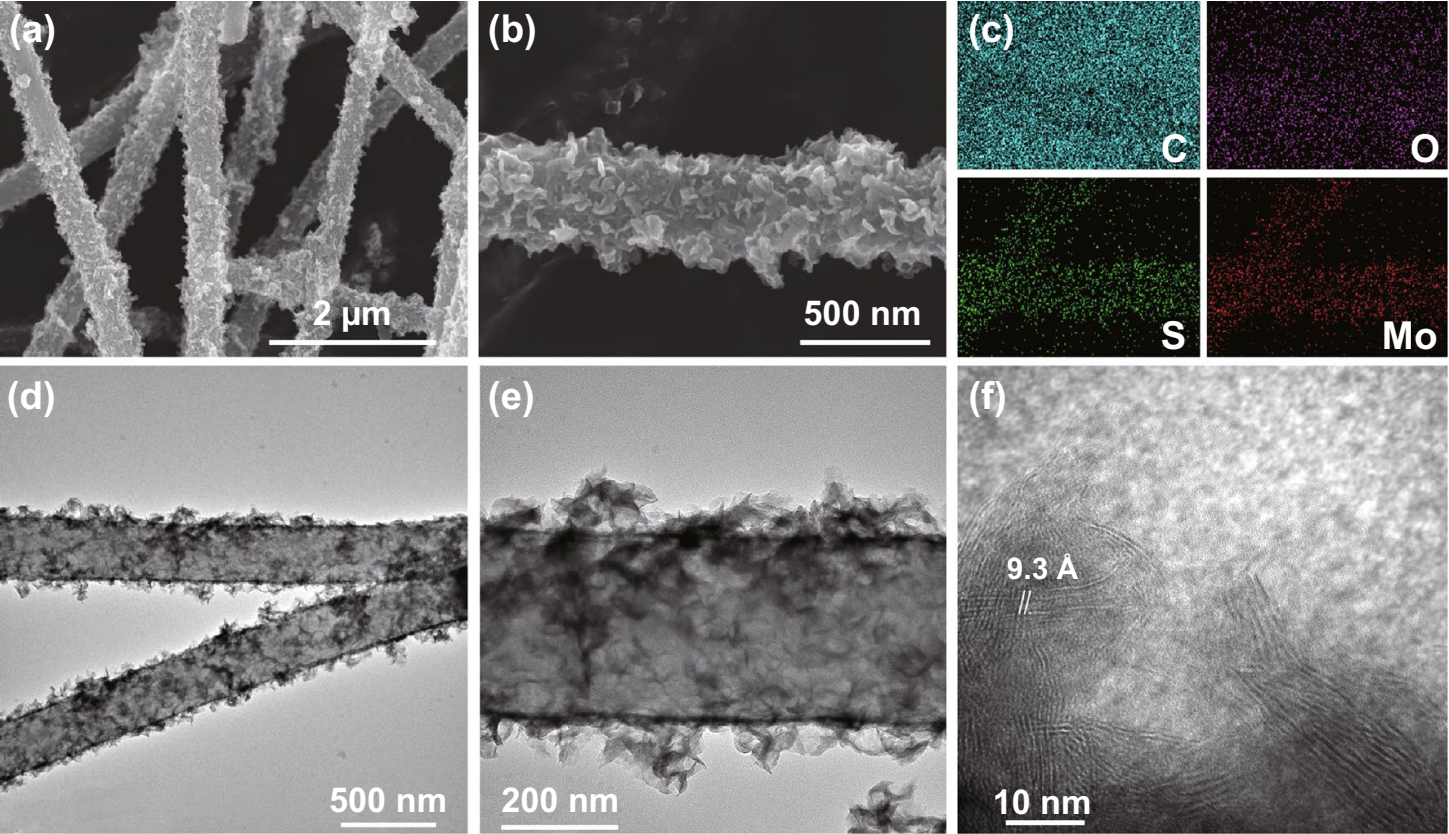
**a**, **b** SEM image, **c** element mapping of C, O, S, Mo, **d**, **e** TEM image, **f** HRTEM images of CF@1T/2H MoS_2_

To prove the microstructure and morphology of the obtained materials even further, SEM and TEM are also used to study the specific information of pure CF, CF@1T/2H MoS_2_, and CF@2H MoS_2_. The bare carbon fibers are made up of countless 400-nm-thin fibers, as shown in Fig. S4. The CF cloth has good flexibility and can be easily bent. After in situ growth of the CF@1T/2H MoS_2_ and CF@2H MoS_2_, it is easily seen that many sheets are coated on the surface of carbon fibers from Figs. 4 and S5, which turns out the same way that we did before to synthesize 1T/2H MoS_2_ and CF@2H MoS_2_ can also be used to synthesize flexible CF-based material. The presence of oxygen element in Fig. 4c comes from CF. Moreover, a large interlayer spacing of 9.3 Å is also observed in Fig. 4f, which should be attributed to the insertion of guest ions or molecules [24].

### Electromagnetic Performance and Parameter

The coaxial transmission line method is adopted with aid of a vector network analyzer to obtain the EM parameters. First, the samples are mixed with paraffin with different mass ratio under 70 °C and then compressed into rings with natural cooling (diameter of the rings: *φ*_ext_ = 7.00 mm, *φ*_int_ = 3.0 mm.) Normally, as for a absorber, the absorption strength mainly depends on the magnetic loss and dielectric loss, which are defined by the complex permeability (*μ*_r_) and permittivity (*ε*_r_) [40–42]:

1 $$\varepsilon_{r} = \varepsilon {^\prime} - j\varepsilon {^\prime }$$

2 $$\mu_{r} = \mu ^{\prime} - j\mu ^{\prime \prime}$$

3 $${\text{tan}}\delta_{\varepsilon } = \varepsilon ^{\prime \prime} /\varepsilon ^{\prime}$$

4 $${\text{tan}}\delta_{\mu } = \mu ^{\prime\prime}/\mu ^{\prime}$$

**Fig. 5:**
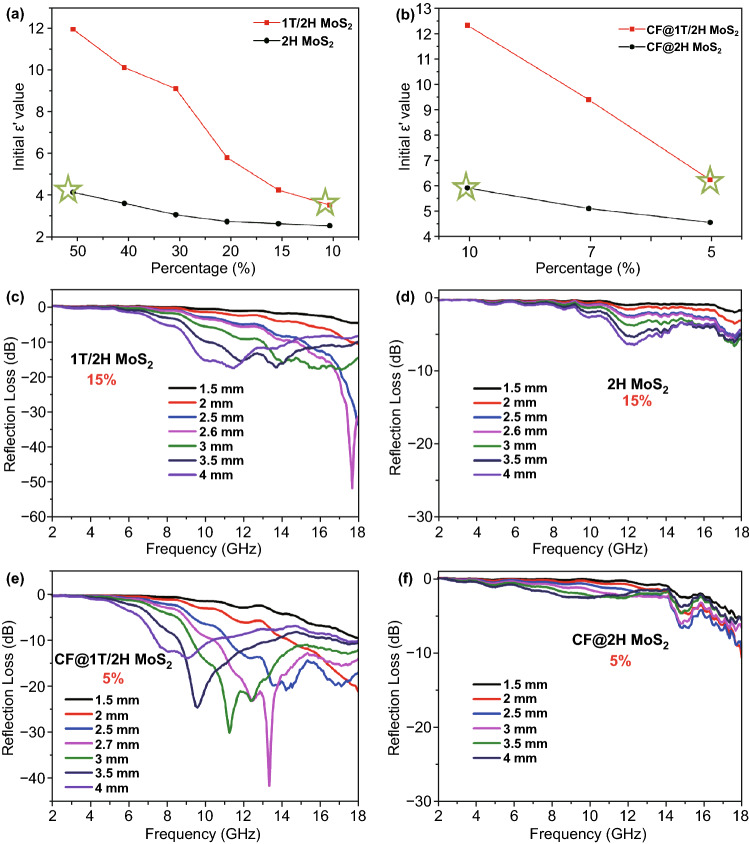
**a**, **b** Matrix loading percentage–initial *ε*′ of 1T/2H MoS_2_ and 2H MoS_2_, CF@1T/2H MoS_2_ and CF@2H MoS_2_. **c**, **d** Calculated reflection loss of 1T/2H MoS_2_ and 2H MoS_2_ with the matrix loading of 15wt%. **e**, **f** CF@1T/2H MoS_2_ and CF@2H MoS_2_ with the matrix loading of 5wt%

Generally speaking, the real and imaginary part indicate energy storage and energy loss, tan*δ*_*ε*_ represents the ratio of energy loss capability to storage capability [43, 44]. In order to effectively explore the influence of different phase on the electromagnetic properties of MoS_2_, we measured the electromagnetic parameters of the 1T/2H MoS_2_ and 2H MoS_2_ with six kinds of proportion; the sample filling mass ratios in sample-paraffin mixture are 50%, 40%, 30%, 20%, 15%, and 10%. As shown in Fig. 5a, as for 1T/2H MoS_2_, the *ε*′ of six ratio 50%, 40%, 30%, 20%, 15%, and 10% are 12, 10.17, 9.16, 5.86, 4.31, and 3.59, respectively. As for 2H MoS_2_, the *ε*′ of six ratios 50%, 40%, 30%, 20%, 15%, and 10% are 4.2, 3.67, 3.13, 2.8, 2.7, and 2.6, respectively. More broadly, all the initial values of two samples both display the downward trend because MoS_2_ is a single dielectric loss type material. Thus, *ε*′ values naturally decrease as the sample proportion decreases. Moreover, the matrix loading percentage—initial *ε*″ curve of 1T/2H MoS_2_ and 2H MoS_2_, is shown in Fig. S6a. As matrix loading percentage goes down, so does initial *ε*″ values. The initial *ε*″ curve of 1T/2H MoS_2_ is still higher than 2H MoS_2_ at the same sample proportion. The all six *ε*′, *ε*″ and tan*δ*_ε_ curves of 1T/2H MoS_2_ and 2H MoS_2_ are shown in Fig. S7. Overall, all *ε*′, *ε*″ and tan*δ*_ε_ values of 1T/2H MoS_2_ are higher than the 2H MoS_2_ under the same sample ratio owing to the high electrical conductivity of 1T/2H MoS_2_. The conductivity of 1T/2H MoS_2_ and 2H MoS_2_ measured by four-point probe is 9 × 10^–2^ and 1.515 × 10^–2^, respectively, as shown in Fig. S9. The high conductivity of 1T/2H MoS_2_ not only can produce the more conduction loss, but also can improve the dielectric property compared with 2H MoS_2._ To be sure, the *μ*′ and *μ*″ values of single dielectric loss type material are the constant value 1 and 0, respectively.

After adding CF, the study sample filling ratio is decreased because CF is also a high-dielectric loss type material. The final study ratios of CF@1T/2H MoS_2_ and CF@2H MoS_2_ are 10%, 7%, 5%. In Fig. 5b, the initial *ε*′ of 10%, 7%, 5% CF@1T/2H MoS_2_ are 12.2, 9.26, 6.1, respectively. Comparatively, the initial *ε*′ of 10%, 7%, 5% CF@2H MoS_2_ are 5.78, 4.97, 4.42, respectively. From Fig. S5b, we can also draw the same conclusion of the initial *ε*″ of CF@1T/2H MoS_2_ with 10%, 7%, and 5% ratios higher than the CF@2H MoS_2_ with 10%, 7%, and 5% ratios. The all three *ε*′, *ε*″, and tan*δ*_ε_ curves of CF@1T/2H MoS_2_ and CF@2H MoS_2_ are shown in Fig. S8a-f. From the result, we can find even the initial *ε*′ value of 10% ratio CF@1T/2H MoS_2_ higher than 50% ratio single 1T/2H MoS_2_, which highlights the role of flexible CF. Furthermore, the conductivity of pure CF, CF@2H MoS_2_, and CF@1T/2H MoS_2_ measured by four-point probe are 1.5 × 10^–1^, 2.257 × 10^–1^, and 5.298 × 10^–1^, respectively, as shown in Fig. S9, consistent with the result of high initial *ε*′ value of 10 wt% CF@1T/2H MoS_2_.

The calculation of reflection loss can be based on the theory of electromagnetic wave transmission line, as shown in the following formula [45–47]:

5 $$R_{{\text{L}}} \left( {d{\text{B}}} \right) = 20\log_{10} \left| {\frac{{Z_{{{\text{in}}}} - Z_{0} }}{{Z_{{{\text{in}}}} + Z_{0} }}} \right|$$

6 $$Z_{{{\text{in}}}} = Z_{0} \sqrt {\mu_{{\text{r}}} /{\upvarepsilon }_{{\text{r}}} } {\text{tan}}h\left[ {j\left( {2\pi fd/c} \right)\sqrt {\mu_{{\text{r}}} \varepsilon_{{\text{r}}} } } \right]$$

Among them, *Z*_in_ refers to the normalized input impedance of electromagnetic wave absorbing materials; *Z*_0_ refers to the impedance matching value in free space; *f* refers to the frequency of incident electromagnetic wave; *d* refers to the thickness of absorbing material; *c* refers to the propagation speed of electromagnetic wave. From Fig. 5c, d, as for the 15% filler loading of single MoS_2_, the more intuitive information can be obtained. As for 1T/2H MoS_2_, when the thickness is 2.6 mm, the minimum reflection loss (*R*_Lmin_) value of 1T/2H MoS_2_ can reach −52.7 dB at 17.7 GHz. The EMW absorption performance of the sample 2H MoS_2_ is shown in Fig. 5d. The 2H MoS_2_ with 15% filler loading is almost impossible to achieve electromagnetic absorption because the low *ε*′ value. Herein, if *R*_L_ = −10 dB at a certain frequency, the material can absorb 90% wave, which can be considered effective absorption. The region *R*_L_ below −10 dB is called effective absorption bandwidth (EAB) [48, 49]. By contrast, the 2H MoS_2_ with high filler loading (50%) can exhibit a good EMW absorbing performance, as shown in Fig. S10. The *R*_Lmin_ is −60 dB when the thickness is 2.8 mm. Though 2H MoS_2_ also can behave effective EMW absorption, it is limited by big filler loading. In general, the 1T/2H MoS_2_ has a better electromagnetic absorption performance.

Figure 5e, f reflects the reflection loss of CF@1T/2H MoS_2_ and CF@2H MoS_2_ with the 5% filler loading. When the thickness is 2.7 mm, the *R*_Lmin_ value of CF@1T/2H MoS_2_ can reach −43 dB at 13.4 GHz only with 5% filler loading. Comparatively, the CF@2H MoS_2_ with 5% filler loading is almost impossible to achieve electromagnetic absorption. This result reasserts the superiority of 1T/2H MoS_2_.

**Fig. 6:**
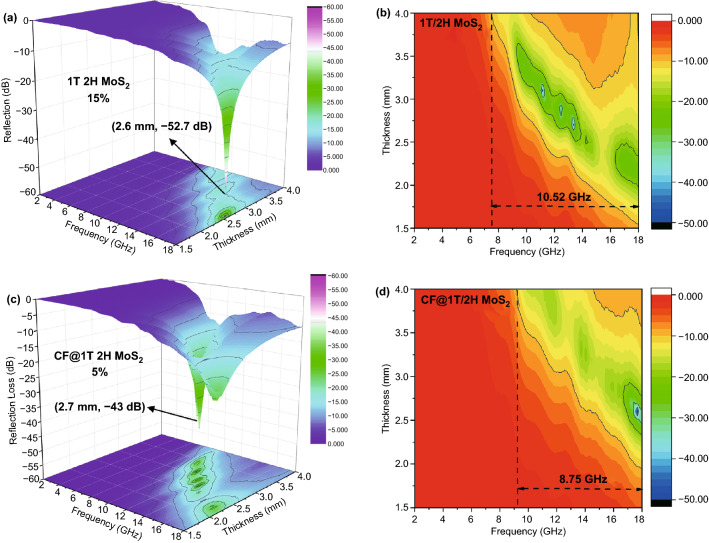
**a**, **c** 3D reflection loss, **b**, **d** corresponding contour maps of 1T/2H MoS_2_ (15%) and CF@1T/2H MoS_2_ (5%)

In order to better reveal the EMW absorbing abilities of different phase, the 3D classical *R*_L_-*f* diagram (Fig. 6) can make a more intuitive comparison between the two kinds of materials. As shown in Fig. 6b, the corresponding EAB of 1T/2H MoS_2_ (15%) can reach 10.52 GHz when the thickness is from 1.5 to 4 mm, which is equivalent to a potential to absorb all waves in the X (8–12 GHz) and Ku (12–18 GHz) bands. As Fig. 6d shows, with the addition of CF, the EAB ranges from 9.25 to 18 GHz.

### EMW Absorption Mechanism

The next step is to explore the electromagnetic absorption mechanism of 1T/2H phase MoS_2_. As we know, good electromagnetic absorbing performance is closely related to the electromagnetic attenuation, more loss (here is dielectric loss) and good impedance matching. Firstly, the propagation paths of incident EMW inside MoS_2_-based absorbers can be enhanced by scattering effect because of the extreme thinness and high specific surface area of MoS_2_. Attenuation matching means the ability and speed to convert the energy of EM waves to other forms of energy. The higher the attenuation constant α is, the closer the material is to attenuation matching [50–52], and the specific formula is as follows:

7 $$\alpha = \frac{\sqrt 2 \pi f}{c} \times \sqrt {\left( {\mu {^{\prime \prime}} \varepsilon {^{\prime \prime}} - \mu {^\prime} \varepsilon {^\prime} } \right) + \sqrt {\left( {\mu {^{\prime \prime}} \varepsilon {^{\prime \prime}} - \mu {^\prime} \varepsilon {^\prime} } \right)^{2} + \left( {\mu {^\prime} \varepsilon {^{\prime \prime}} + \mu {^{\prime \prime}} \varepsilon {^\prime} } \right)^{2} } }$$

**Fig. 7:**
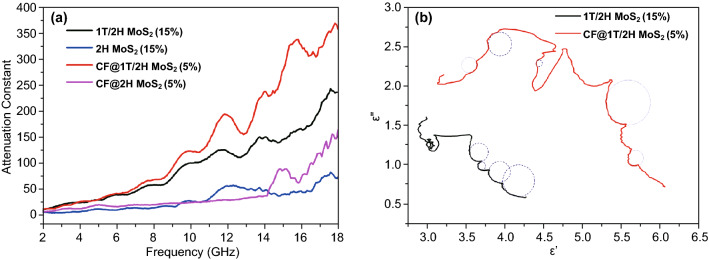
**a** Attenuation constant of 1T/2H MoS_2_ and 2H MoS_2_ (15%), CF@1T/2H MoS_2_ and CF@2H MoS_2_ (5%). **b** Cole–Cole semicircle of 1T/2H MoS_2_ (15%) and CF@1T/2H MoS_2_ (5%)

In Fig. 7a, four curves rise by frequency, and with the addition of CF, all the curves are in order of height: CF@1T/2H MoS_2_ (5%) ≥ 1T/2H MoS_2_ (15%) ≥ CF@2H MoS_2_ (5%) ≥ 2H MoS_2_ (15%). The maximum α value rises from 50 to 350. At their *R*_Lmin_ point, α of CF@1T/2H MoS_2_ (5%) is 236, larger than that of the CF@2H MoS_2_ (5%). The α of 1T/2H MoS_2_ (15%) is 73, still larger than that of the 2H MoS_2_ (15%). This proves that the 1T/2H MoS_2_ based material has good attenuation loss capacity.

The dielectric loss in 2–18 GHz is chiefly dominated by polarization relaxation. Figure 7b shows the Cole–Cole semicircle analyzing how many relaxation processes each material own. According to the Debye theory,

8 $$\left( {\varepsilon {^\prime} - \frac{{\varepsilon_{{\text{s}}} + \varepsilon_{\infty } }}{2}} \right)^{2} + \left( {\varepsilon {^{\prime\prime}} } \right)^{2} = \left( {\frac{{\varepsilon_{{\text{s}}} - \varepsilon_{\infty } }}{2}} \right)^{2}$$

When *ε*″ varies with *ε*′, every semicircle represents a relaxation process [53–55]. The arc could be regarded as a similar process. For the 1T/2H MoS_2_, four processes are distinguished, while the CF@1T/2H MoS_2_ has five. More processes supply more dielectric loss; thus, the CF@1T/2H MoS_2_ has better dissipation ability.

To achieve an excellent absorption performance, the prerequisite is the less reflection, and zero reflection of the incident microwave is the best. Based on the transmission line theory, if the minimum *R*_L_ could correspond to impedance matching ratio value (*Z* =|*Z*_in_/*Z*_0_|) equal 1 at the same frequency, the impedance of this material matches well. The calculation formula is as follows [56–59]:

9 $$Z_{{{\text{in}}}} = Z_{0} \sqrt {\mu_{{\text{r}}} /{\upvarepsilon }_{{\text{r}}} } {\text{tan}}h\left[ {j\left( {2\pi fd/c} \right)\sqrt {\mu_{{\text{r}}} \varepsilon_{{\text{r}}} } } \right]$$

**Fig. 8:**
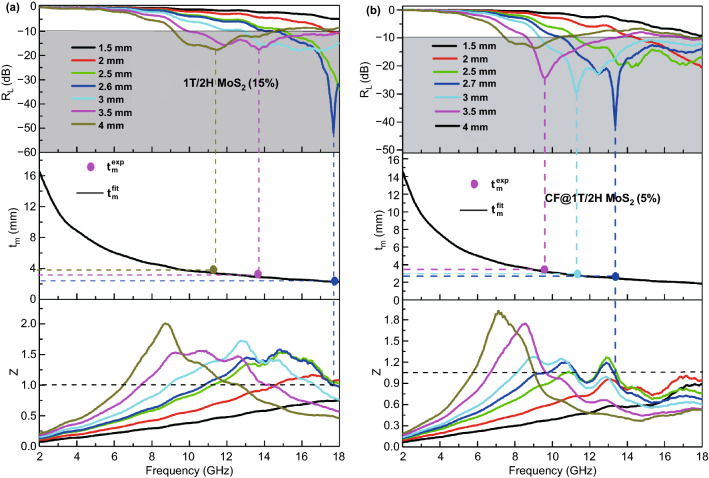
Dependence of the matching thickness (*t*_m_) on frequency (*f*_m_) under λ/4 and normalized input impedance of 1T/2H MoS_2_ (15%) and CF@1T/2H MoS_2_ (5%)

The image in Fig. 8 displays the normalized input impedance of as-prepared samples. As we can see, the CF@1T/2H MoS_2_ (2.7 mm) and 1T/2H MoS_2_ (2.6 mm) meet this requirement, demonstrating the good impedance matching of 1T/2H MoS_2_-based absorber.

When the thickness of the material is increasing, the frequency corresponding to minimum *R*_L_ becomes smaller. This regularity is in line with the ¼ wave length model as follows [60–62]:

10 $$t_{{\text{m}}} = \frac{nc}{{4f_{{\text{m}}} \sqrt {\left| {\mu_{r} } \right|\left| {{\upvarepsilon }_{r} } \right|} }}$$

When the value of *t*_m_^exp^ just falls on the curve *t*_m_^fit^, the electromagnetic wave is canceled because the two reflected waves formed by air-absorber and the metal-absorption interface form 180° out of phase. In the middle image of Fig. 8, all two curves of fit thickness decline with the increase in frequency. The circle marks correspond to the frequency that *R*_L_ is reaching the minimum. The 1T/2H MoS_2_ (15%) and CF@1T/2H MoS_2_ (5%) perfectly fit the model by realizing the equality of two thicknesses of 2.6 and 2.7 mm, respectively. In total, considering the high dielectric loss result from better conductivity, big attenuation, good impedance matching and low filler loading, the as-prepared 1T/2H MoS_2_ is expected to exhibit excellent EMW absorbing abilities. Compared with the previous MoS_2_-based EMW absorbers, as shown in Table 1, the 1T/2H MoS_2_ and CF@1T/2H MoS_2_ can achieve the effective electromagnetic absorption only with low filler loading (15%) and (5%), respectively.

**Table 1:** Summary of MoS_2_-based EMW absorbers

Material	Ratio	Thickness (mm)	EAB (GHz) (R_L_ ≤ −10 dB)	*R*_Lmin_ (dB)	Refs.
PB@ MoS_2_	40%	2.4	10.2	−42.83	[14]
CNTs@MoS_2_	40%	1.5	4	−35	[15]
CoFe_2_O_4_@1T/2H-MoS_2_	40%	1.81	14.5	−68.5	[27]
MoS_2_	60%	2.4	7.44	−47.8	[28]
MoS_2_-NS	60%	2.4	9.36	−38.42	[29]
1T/2H MoS_2_@RGO	30%	2.5	4	−67.77	[31]
1T/2H MoS_2_ work	15%	2.6	10.52	−52.7	This work
CF@1T/2H MoS_2_	5%	2.7	8.75	−43	This work

### Radar Cross Section

**Fig. 9:**
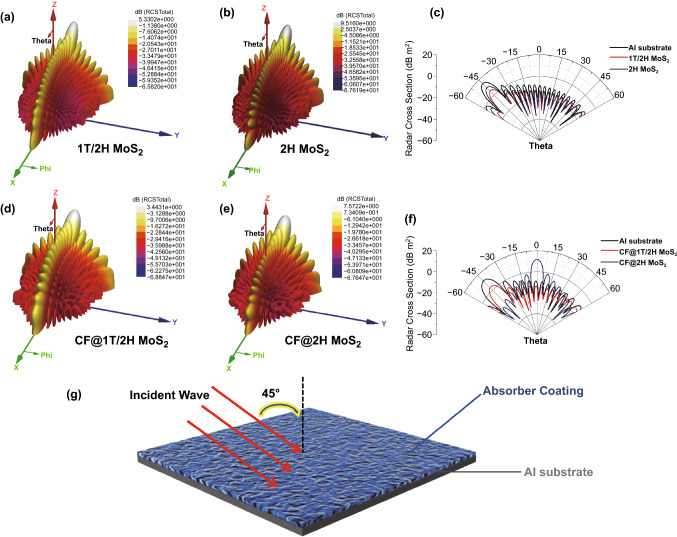
**a**, **b**, **d**, **e** Three-dimensional spherical coordinate diagrams. **c**, **f** Polar coordinate diagram. **g** Schematic diagram of HFSS simulation analysis

When the geometrical shape of the absorbing material is stable, the radar cross section (RCS) is an important index to judge the absorbing ability of the absorbing material [63, 64]. The HFSS simulation is used to explore the RCS performance of CF@1T/2H MoS_2_ and 1T/2H MoS_2_. The aluminum (Al) plate is used as the substrate and set to a thin tube with 180 mm long and 5 mm thick [65]. The prepared samples are mixed with paraffin as the absorber coating; it has the same length as the Al plate but the thickness is chosen the above calculated thickness value corresponding to the optimum EMW absorbing performance. In this work, the thickness of 1T/2H MoS_2_ is 2.6 mm, the thickness of CF@1T/2H MoS_2_ is 2.7 mm, and the schematic diagram is shown in Fig. 9g. The incident direction of EM wave is oblique at 45°, the calculation can begin when the material is given an appropriate excitation boundary. After simulation calculations, the RCS values of Al plate and different absorbers are obtained. The 3D spherical coordinate diagrams in different directions and polar plots between −60°–60° are shown in Fig. 9a–f. It can be seen that when the EMW is incident on the Al plate coated with MoS_2_ absorber/paraffin mixture, the RCS values are smaller than that of single Al plate. In particular, the RCS values of 1T/2H MoS_2_ are smaller than 2H MoS_2_, which further proves the superiority of 1T/2H MoS_2_ as electromagnetic wave absorber. As for CF@1T/2H MoS_2_ and CF@2H MoS_2_, we can also draw a conclusion that the RCS values of CF@1T/2H MoS_2_ are smaller than CF@2H MoS_2_. In summary, the absorbers of 1T/2H MoS_2_ based material exhibit excellent EMW absorption performance.

## Conclusion

In this work, we successfully synthesize the 1T/2H MoS_2_ and 2H MoS_2_ through a facile hydrothermal route and profoundly explore the influence of different MoS_2_ phase for electromagnetic absorbing properties by analyzing electromagnetic parameters of 1T/2H MoS_2_ and 2H MoS_2_ with 50%, 40%, 30%, 20%, 15%, and 10% filler loading. As a result, the *R*_Lmin_ of 1T/2H MoS_2_ only with 15% filler loading can reach −52.7 dB at 17.7 GHz when the thickness is 2.6 mm. The excellent EMW absorption performance of 1T/2H MoS_2_ than 2H MoS_2_ is due to the high dielectric loss result from better conductivity, big attenuation and good impedance matching. In addition, taking the advantage of 1T/2H MoS_2_, the flexible CF@1T/2H MoS_2_ is also synthesized to mind the request of flexible portable microwave absorption electronic devices. When the thickness is 2.7 mm, the *R*_Lmin_ value of CF@1T/2H MoS_2_ can reach −43 dB at 13.4 GHz only with 5% filler loading.

## Supplementary Information

Below is the link to the electronic supplementary material.

Supplementary file1 (PDF 1906 kb)
