# Supplementary material for: MoS2-Decorated/Integrated Carbon Fiber: Phase Engineering Well-Regulated Microwave Absorber
Source: Nanomicro Lett. 2021 Apr 27;13:114. doi: 10.1007/s40820-021-00646-y (PMC8079512; doi:10.1007/s40820-021-00646-y)
Supplement: Supplementary file 1 — Supplementary file1 (PDF 1906 kb) [file 40820_2021_646_MOESM1_ESM.pdf]

Supporting Information for

MoS_2 Decorated/Integrated Carbon Fiber: Phase Engineering Well-Regulated Microwave Absorber

Jing Yan[1], Ying Huang[1,]*, Xiangyong Zhang[2], Xin Gong[3], Chen Chen[1], Guangdi Nie[4], Xudong Liu[1], Panbo Liu[1]

[1]MOE Key Laboratory of Material Physics and Chemistry under Extraodinary Conditions, Ministry of Education, School of Chemistry and Chemical Engineering, Northwestern Polytechnical University, Xi'an 710072, P. R. China

[2]School of Materials Science and Engineering, Central South University, Changsha 410083, P. R. China

[3]Institute of Flexible Electronics, Northwestern Polytechnical University, Xi'an 710072, P. R. China

[4]Industrial Research Institute of Nonwovens & Technical Textiles, College of Textiles and Clothing, Qingdao University, Qingdao 266071, P. R. China

*Corresponding author. E-mail: yingh@nwpu.edu.cn (Ying Huang)

S1 Supplementary Experiment

S1.1 Synthesis of CF

Typically, a 10 wt% precursor solution was obtained by mixing 0.5 g of PAN with 4.5 g of DMF at 60 °C, which was stirred for about 2 h. The specific electrospinning parameters as follow, collecting distance is ~18 cm, applied voltage is 18 kV, and solution feed rate is 12 μL min^{-1}. The resultant PAN nanofibers were finally pre-oxidized at 260 °C (heating rate: 2 °C min^{-1}) in air for 2 h and then carbonized at 900 °C (heating rate: 5 °C min^{-1}) in Ar flow for another 2 h.

S1.2 Characterization

A field-emission scanning electron microscope (FESEM, Verios G4) and a transmission electron microscope (TEM, JEOL 2010 transmission electron microscope) were used to observe the morphology and size of the particles, respectively. X-ray powder diffraction (XRD) measurements were performed on a Bruker D2Phaser X-ray diffractometer with Cu Kα radiation (λ = 1.5418 Å). The specific structural characteristics of MoS_2 were characterized by a Raman spectrometer (WITec Alpha300R; λ = 514 nm). The element composition and chemical binding state of the samples were determined by X-ray photoelectron spectroscopy (XPS; Phoibos 100 spectrometer). The conductivity of the material is measured by an SX1994 four-point probe meter. The electromagnetic parameters were measured by a vector network analyzer (Agilent E5071C; coaxial method) in the

range of 2-18 GHz. The 1T/2H MoS_2 and 2H MoS_2 were mixed with paraffin at 50wt%, 40wt%, 30wt%, 20wt%, 15wt% and 10wt% to make a coaxial ring (external diameter, 7.0 mm; internal diameter, 3.0 mm; H, 2.5 ± 0.5 mm). Moreover, the CF@1T/2H MoS_2 and CF@2H MoS_2 were mixed with paraffin at 10 wt%, 7 wt% and 5wt%.

S2 Supplementary Figures

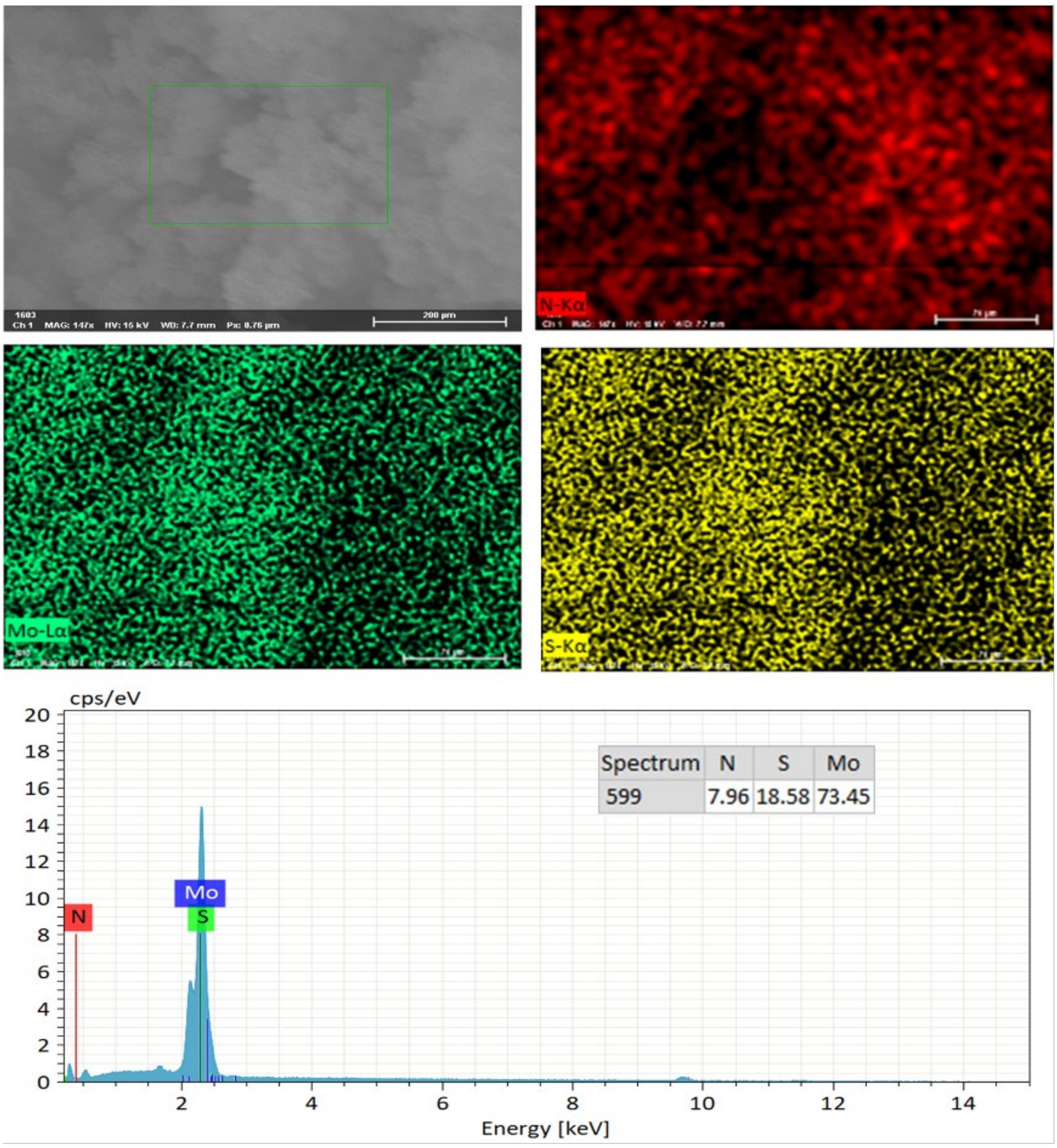

Fig. S1 Element mapping images and EDX of 1T/2H MoS_2 (the EDX of 1T/2H MoS_2 only can prove the presence of N, the ratio of S to Mo is not accurate because the location is too close)

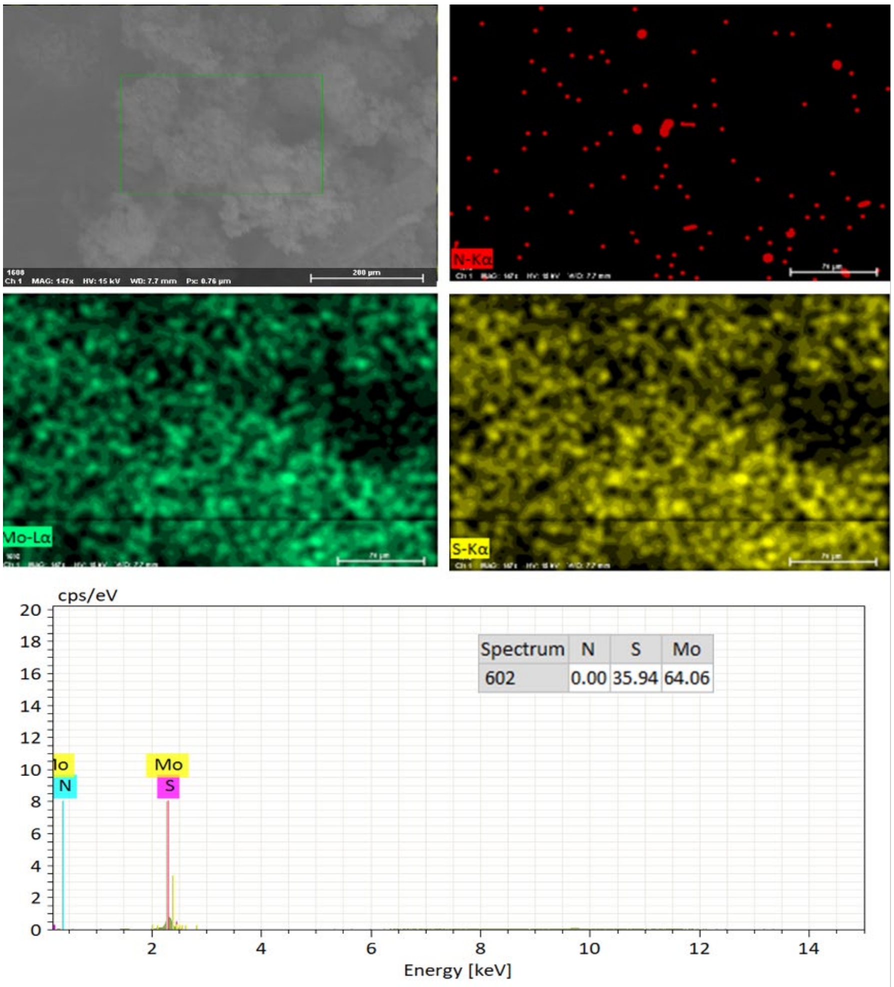

Fig. S2 Element mapping images and EDX of 2H MoS_2 (the EDX of 2H MoS_2 only can prove the absence of N to compare with 1T/2H MoS_2, the ratio of S to Mo is not accurate because the location is too close)

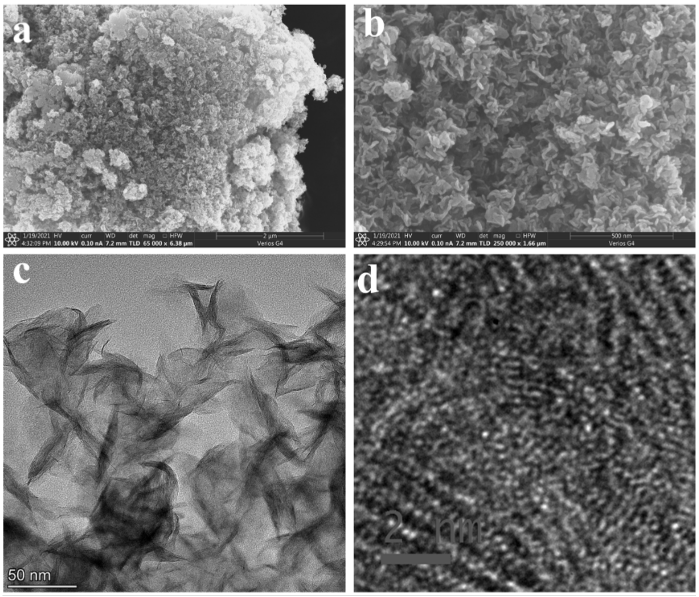

Fig. S3 a-b SEM images. **c** TEM images. **d** HRTEM of 2H MoS_2

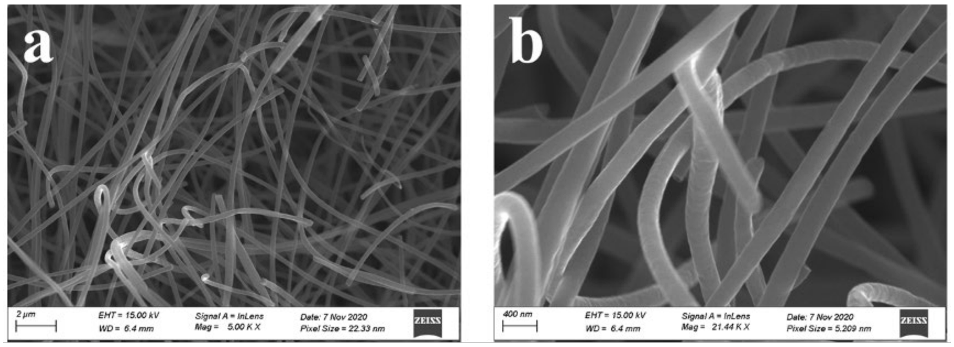

Fig. S4 a-b SEM images of CF

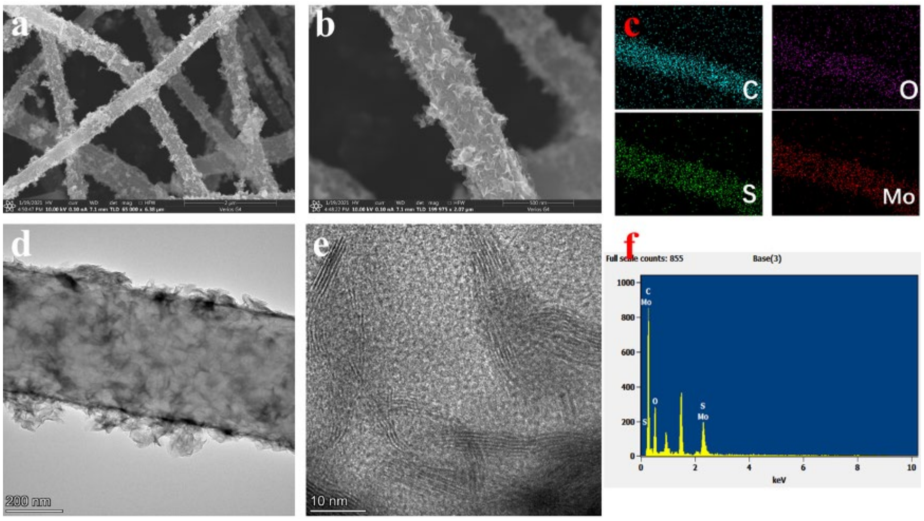

Fig. S5 a-b SEM images. **c** element mapping of C, O, S, Mo. **d** TEM images. **e** HRTEM. **f** EDX of CF@2H MoS_2

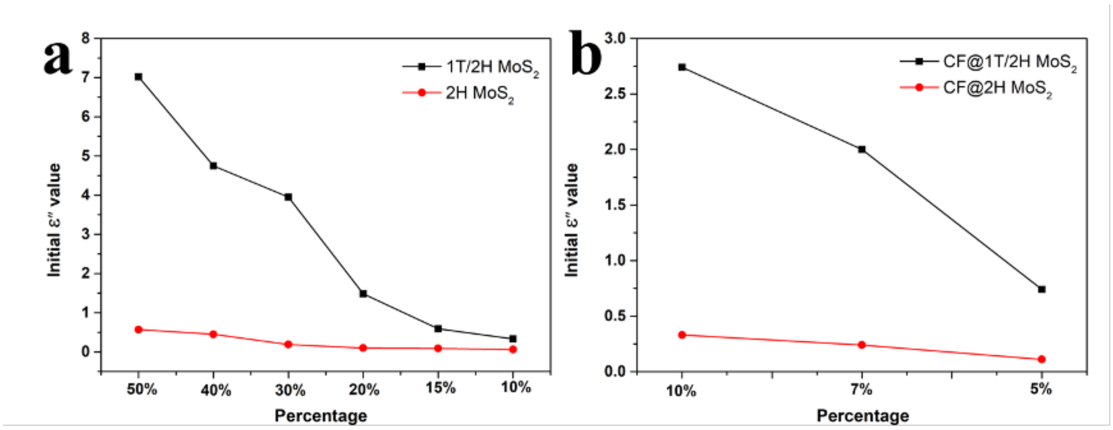

Fig. S6 a-b Matrix loading percentage-initial ε″ of 1T/2H MoS_2 and 2H MoS_2, CF@1T/2H MoS_2 and CF@2H MoS_2

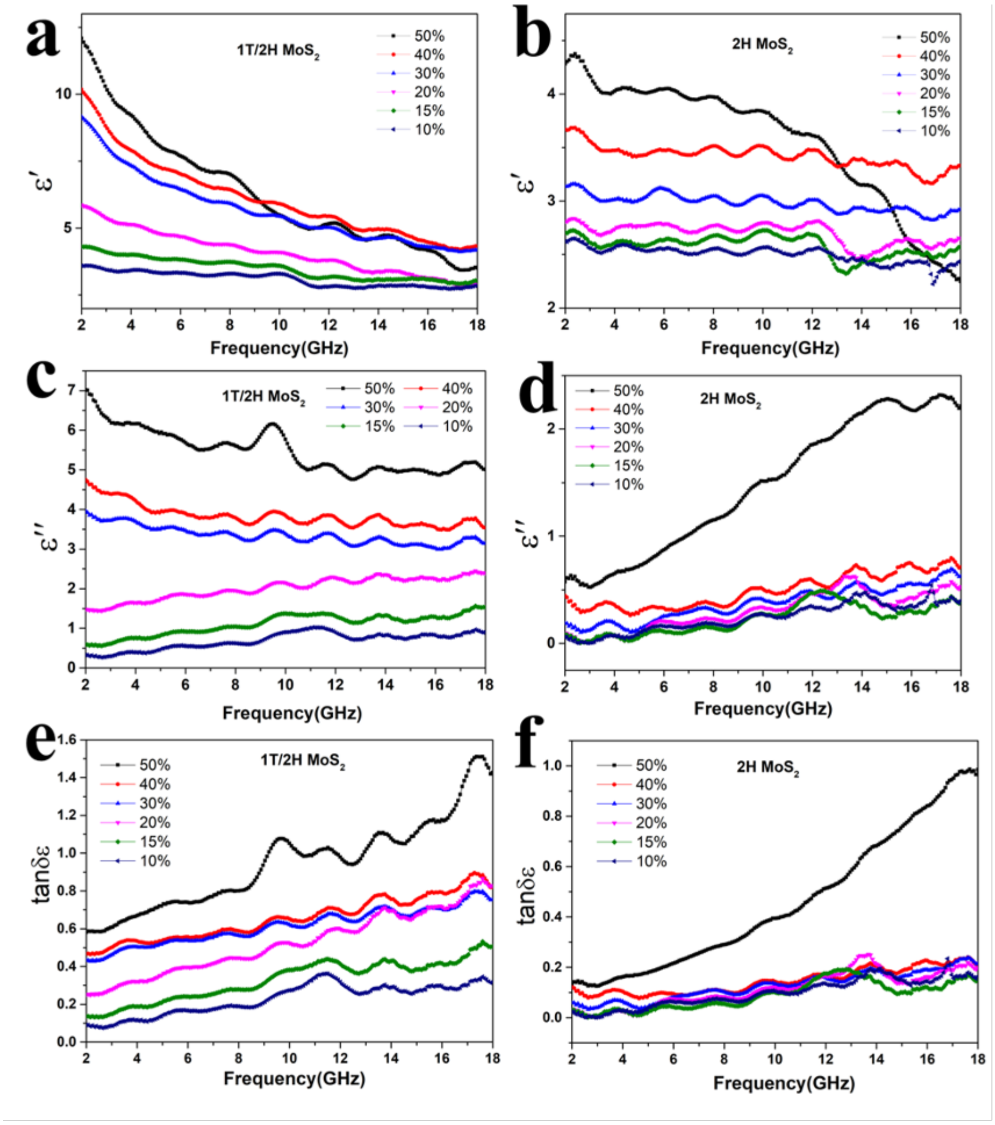

Fig. S7 a, c, e ε′, ε″ and tanδ_ε of 1T/2H MoS_2. **b, d, f** ε′, ε″ and tanδ_ε of 2H MoS_2 with the matrix loading of 50wt%, 40wt%, 30wt%, 20wt%, 15wt% and 10wt%

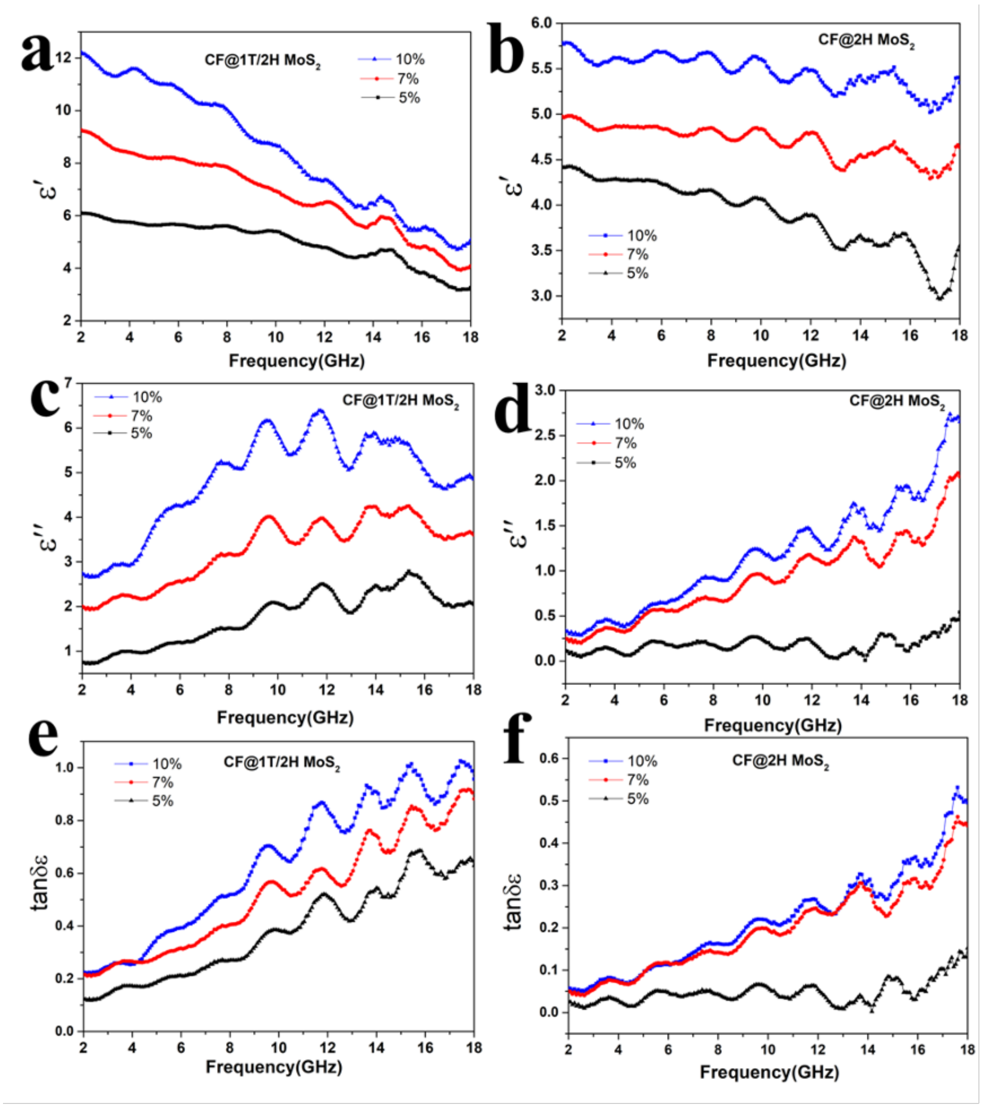

Fig. S8 a, c, e ε′, ε″ and $\tan\delta_\varepsilon$ of CF@1T/2H MoS_2, **b, d, f** ε′, ε″ and $\tan\delta_\varepsilon$ of CF@2H MoS_2 with the matrix loading of 10wt%, 7wt% and 5wt%

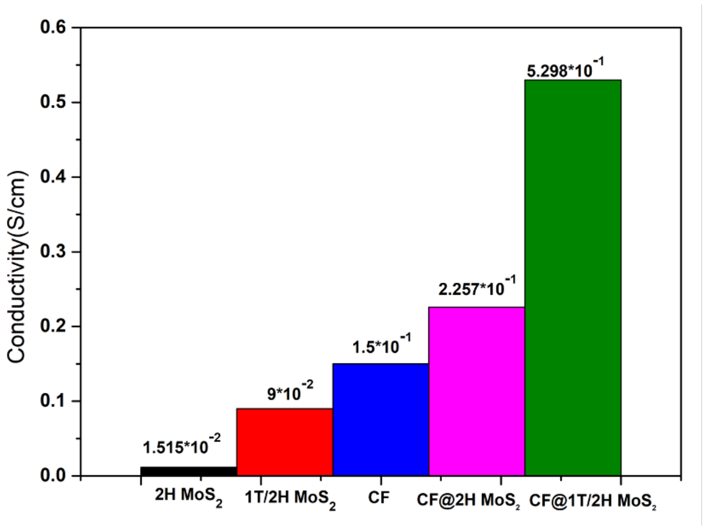

Fig. S9 Conductivity comparison of five samples

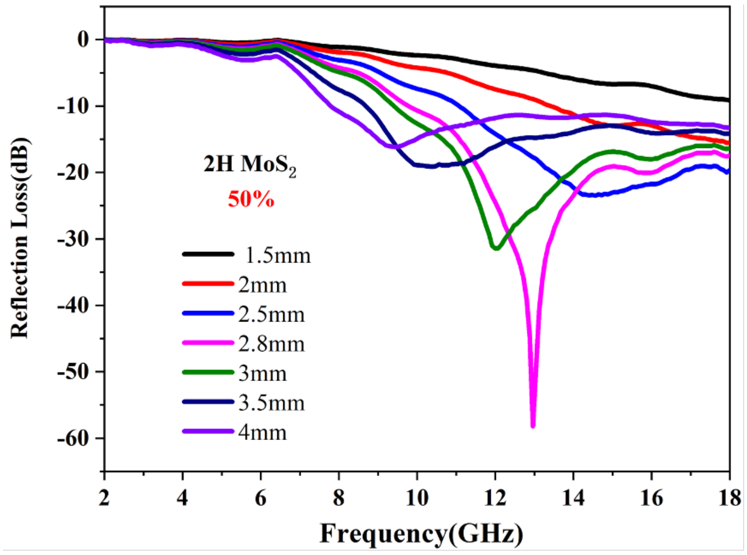

Fig. S10 Calculated reflection loss of 2H MoS_2 with the matrix loading of 50wt%
